# Supplementary material for: An open-source framework for large-scale, flexible evaluation of biomedical text mining systems
Source: J Biomed Discov Collab. 2008 Jan 29;3:1. doi: 10.1186/1747-5333-3-1 (PMC2276192; doi:10.1186/1747-5333-3-1)
Supplement: Additional file 1 — Evaluation platform source code. The additional file contains the software evaluation framework discussed in this paper. The most current release can be downloaded from SourceForge.net [1]. See the accompanying README file for instructions on installation and use. Also included in the distribution is a collection of UIMA wrappers for some commonly used BioNLP tools and annotated corpora. [file 1747-5333-3-1-S1.zip › bionlp_uima_repository-1.0.1/docs/api/edu/uchsc/ccp/uima/ae/nlp/opendmap/CCPOpenDMAP_AE.html]

CCPOpenDMAP\_AE (Center for Computational Pharmacology Util/UIMA API)


|  |  |  |
| --- | --- | --- |
| **Overview** \| **Package** \| **Class** \| **Tree** \| **Deprecated** \| **Index** \| **Help** | |  |
| PREV CLASS   **NEXT CLASS** | **FRAMES**    **NO FRAMES**     **All Classes** |  |
| SUMMARY: NESTED \| FIELD \| CONSTR \| METHOD | DETAIL: FIELD \| CONSTR \| METHOD |  |


---


## edu.uchsc.ccp.uima.ae.nlp.opendmap Class CCPOpenDMAP\_AE

```
java.lang.Object
  org.apache.uima.analysis_engine.annotator.Annotator_ImplBase
      org.apache.uima.analysis_engine.annotator.JTextAnnotator_ImplBase
          edu.uchsc.ccp.uima.ae.nlp.opendmap.OpenDmapAnnotator
              edu.uchsc.ccp.uima.ae.nlp.opendmap.CCPOpenDMAP_AE
```

**All Implemented Interfaces:**: org.apache.uima.analysis\_engine.annotator.BaseAnnotator, org.apache.uima.analysis\_engine.annotator.JTextAnnotator

---

``` public class CCPOpenDMAP_AE extends OpenDmapAnnotator ```

This class packages OpenDMAP into the UIMA framework.

This annotator assumes that the input GeneRIF can be found in
the JCas in CCPTextAnnotation structures and copies the output
references of interest into the JCas as CCPTextAnntotion
structures.

**Author:**
:   R. James Firby

---

**Constructor Summary**

`CCPOpenDMAP_AE()`
  
          Create a new GeneRIF Annotator that uses OpenDMAP to
process the input document text.


| **Method Summary** | |
| --- | --- |
| `void` | `initialize(org.apache.uima.analysis_engine.annotator.AnnotatorContext context)`             Initialize the GeneRIF Annotator. |
| `void` | `process(edu.uchsc.ccp.opendmap.Parser parser, org.apache.uima.jcas.JCas jcas, org.apache.uima.analysis_engine.ResultSpecification resultSpec)`             Process the JCas using the annotation map to help extract input tokens. |

**Methods inherited from class edu.uchsc.ccp.uima.ae.nlp.opendmap.OpenDmapAnnotator**

`addPatternFilename, process, setProtegeProjectFilename`

**Methods inherited from class org.apache.uima.analysis\_engine.annotator.Annotator\_ImplBase**

`destroy, finalize, getContext, getTypeSystem, reconfigure, typeSystemInit`

**Methods inherited from class java.lang.Object**

`clone, equals, getClass, hashCode, notify, notifyAll, toString, wait, wait, wait`

**Methods inherited from interface org.apache.uima.analysis\_engine.annotator.BaseAnnotator**

`destroy, reconfigure, typeSystemInit`

**Constructor Detail**

### CCPOpenDMAP\_AE

```
public CCPOpenDMAP_AE()
```

:   Create a new GeneRIF Annotator that uses OpenDMAP to
    process the input document text.


**Method Detail**

### initialize

```
public void initialize(org.apache.uima.analysis_engine.annotator.AnnotatorContext context)
                throws org.apache.uima.analysis_engine.annotator.AnnotatorInitializationException
```

:   Initialize the GeneRIF Annotator. Get the parameter values, load
    the annotation map, and create the parser.

    :   **Specified by:**: `initialize` in interface `org.apache.uima.analysis_engine.annotator.BaseAnnotator` **Overrides:**: `initialize` in class `OpenDmapAnnotator`
    :   **Throws:**: `org.apache.uima.analysis_engine.annotator.AnnotatorInitializationException`

---


### process

```
public void process(edu.uchsc.ccp.opendmap.Parser parser,
                    org.apache.uima.jcas.JCas jcas,
                    org.apache.uima.analysis_engine.ResultSpecification resultSpec)
```

:   Process the JCas using the annotation map to help extract input tokens.
    Write the references found back into the JCas using the annotation map to
    describe the desired output annotations.

    :   **Specified by:**: `process` in class `OpenDmapAnnotator`
    :   **Parameters:**: `parser` - The OpenDMAP parser to use for parsing.: `jcas` - The JCas holding input tokens and output annotations.: `resultSpec` - The type of result expected to be put into the JCas.


---


|  |  |  |
| --- | --- | --- |
| **Overview** \| **Package** \| **Class** \| **Tree** \| **Deprecated** \| **Index** \| **Help** | |  |
| PREV CLASS   **NEXT CLASS** | **FRAMES**    **NO FRAMES**     **All Classes** |  |
| SUMMARY: NESTED \| FIELD \| CONSTR \| METHOD | DETAIL: FIELD \| CONSTR \| METHOD |  |


---
